# Editorial: Investigating the Impact of Current Issues on Leisure, Tourism, and Hospitality in Psychological Science

**DOI:** 10.3389/fpsyg.2020.596868

**Published:** 2020-11-27

**Authors:** Anestis K. Fotiadis, Chris A. Vasilliadis, Tzung-Cheng Huan

**Affiliations:** ^1^Department of Management, College of Business, Zayed University, Abu Dhabi, United Arab Emirates; ^2^Department of Business Administration, School of Business Administration, University of Macedonia, Thessaloniki, Greece; ^3^National Chiayi University and Tainan University of Technology, Tainan, Taiwan

**Keywords:** current issues, tourism, hospitality, psychology, leisure

As one of the world's most dynamic, fast-changing, and intense industries, tourism remains the primary engine of economic growth and sustainability generating a vast number of employment opportunities leading to poverty alleviation (Fotiadis et al., 2019a). Current challenges occurring from the Covid-19 pandemic indicate that the tourism and hospitality sectors are highly sensitive to changes and are crucial for the global economy (Polyzos et al., 2020; Shehzad et al., 2020). Psychological impacts of financial distress are a significant field of study. Usually, a financial crisis will create direct or indirect micro and macro impacts in different countries and environments.

The tourism industry is a complex environment comprising different sectors with their complex environments. Thereby, any change, be it a minor or major one, may have a significantly positive or negative impact on stakeholders. For that reason, community-based tourism is an emerging field, and growing numbers of studies are examining resident's behaviors and how development can be beneficial—or not—for a local community (Knez and Eliasson, 2017). Local communities tend to be more active when destination leadership is stable and innovative (Bichler, 2019). As Farmaki (2021) designates, special care should be used in post-crisis experiences, especially for the local community, as small local communities are unable to react easily to significant changes (Fotiadis et al., 2019b).

In recent years, the rise of medical tourism is an example that reshapes the tourism industry based on the fast-growing segments of retired tourists with their expectations regarding the provision and delivery of tourism products and services. As Garcia-Garzon et al. (2016) mention, medical travel has grown significantly during recent years, supporting new markets and advancing medical care. As expected, the recent pandemic transforms everything we knew so far and created a new tourism model where new types of medical tourism development are needed.

This special issue seeks to shed light on the current academic and practical perspectives within the leisure, tourism, and hospitality sectors. More so, this special issue investigates contemporary concerns relative to leisure, tourism, and hospitality to develop new theoretical constructs and perspectives, and stimulate dialogues in this respect while trying to strike a balance between theory and application. In this end, this special issue invited offerings from various disciplines in aiming to serve as a forum through which these various disciplines may interact and thereby expand the body of literature on leisure, tourism and hospitality, and social science at large. The relationship between psychological science and new methods used in the tourism industry, such as neuroscience, artificial intelligence, and big data analysis, can offer better insights into the new era tourism and hospitality industry.

As such, we briefly present the papers included in this issue.

The first paper, entitled “How to promote ethnic village residents' behavior participating in tourism poverty alleviation: A tourism empowerment perspective,” is authored by Yang et al.. This study examines tourism empowerment's effect on local village inhabitants' behaviors contributing to tourism poverty mitigation in Zenlei Village in Sandu County of Guizhou Province in China. The authors develop four hypotheses related to the “Tourism Empowerment and Participation Behavior” subtheme. Then they examine four hypotheses in the mediating role of participation willingness in relation to tourism empowerment and participation behavior. Furthermore, they assess four more hypotheses to examine the moderating role of participation ability regarding tourism empowerment and participation behavior. As the results delineate, tourism empowerment has a significant interrelation with participation behavior, while there is a mediating effect among participation willingness, tourism empowerment, and participation behavior. Additionally, it is demonstrated that a strong positive correlation exists among tourism psychological empowerment and participation willingness when residents' participation ability is high and weak when it is low.

The second paper is titled “Evaluation of self-assessed state of health and vitamin D knowledge in Emirati and international female students in United Arab Emirates (UAE)” and is authored by Abboud et al.. In this paper, the authors compare knowledge about vitamin D and the perceived state of health in Emirati and international female tourism students in Dubai, UAE. Their study focuses on a niche market in the middle east, as they are exploring female Emiratis and international female tourists. Their research questions explore how different dietary variables, such as assessed levels of supplementation, diet, and UV exposure, can affect the perceived state of health. In addition, they evaluated the participants' self-assessed state of health in terms of vitamin D testing and general well-being indicators. As their results signify, there is an exceedingly low knowledge about the association of vitamin D deficiency and most diseases. Further, Emirati students reported using Vitamin D supplements much more than the international students.

The third paper deals with “Resident's perspective on developing community-based tourism—A qualitative study of Muen Ngoen Kong community, Chiang Mai, Thailand,” authored by Lo and Janta. This study examines the benefits and challenges of Community-Based Tourism (CBT) and solutions to address identified shortcomings by studying the Muen Ngoen Kong community in Chiang Mai, Thailand. Focusing on the concept of CBT, the importance of CBT, and CBT development's objectives, the researchers developed three research questions. The first one explores the challenges a community faces concerning CBT. The second one investigates benefits that Support CBT Development in the Community, while the third suggests solutions to solve the challenges. Results designate that there are many problems with how CBT is implemented. The most significant problems seem to be resource ownership, benefit leaking, financial issues, and limited community participation.

The fourth paper is titled “Emirati adults have a higher overall knowledge on vitamin D compared to Tourists” and is authored by Saleh et al.. This research examines the level of knowledge of vitamin D, calcium, and physical activity among Emirati and tourist adults in Abu Dhabi. It is a cross-sectional study undertaken in three different malls in Abu Dhabi, where the retail and hospitality sectors are well-developed and include Emirati and tourist patrons. Another research question investigates if there are demographic differences in perceptions. Emirati participants showed a higher overall vitamin D knowledge than their tourist counterparts. Both groups indicated a low/medium level of knowledge regarding physical activity, calcium and vitamin D supplements.

The fifth research paper is a brief report titled “Promises and hurdles of medical tourism development in the russian federation,” where Daykhes et al. are the authors of this study. The primary purpose of this research was to identify factors affecting the development of medical tourism in Russia and those factors that impede this area's development using the expert assessment method. The authors surveyed the complex relationships that might exist between the health expenditure landscape and medical tourism. Another investigation area was to determine the main issues that impede the medical tourism field in Russia and suggest possible solutions for further development. They develop 10 research questions regarding the perception of foreign medical tourists, and their results suggest nine improvements for further progressing the industry in Russia.

The next study deals with the topic “Exploring consumers' behavior for choosing sustainable food,” authored by Hsu et al.. In their study, the authors explore consumers' interests in buying sustainable food in Taiwan. The study focused on interest instead of intention or behavior to better understand the formation of interest. For their study, the authors develop four hypotheses. The first one is examining the relationship between knowledge and interest in buying sustainable food. The second examines friends' support regarding the level of interest, while the third addresses the relationship between price and interest. In the final hypothesis, they explore health incentives and levels of interest. The results indicate that the first three hypotheses were supported while the fourth was not.

Research paper number seven is titled “Behavioral model of middle-aged and seniors for bicycle tourism.” The authors of this study are Lin et al.. Their study seeks to determine the behavioral tendency of the middle-aged and seniors in bicycle tourism at environmentally protected scenic areas and its relevant influence factors. The authors use the theory of planned behavior (TPB) and develop six hypotheses. The first three hypotheses examine the relationship among sports habits and subjective norms, perceived behaviors, and respondents' attitudes toward biking. The other three examine the association of respondent's behavioral intentions and attitudes, subjective norms, and perceived behaviors. A structural equation model was developed, and each hypothesis was accepted.

The next paper is entitled “A study on the place attachment of golf club members,” authored by Chen et al.. In this paper, the authors explore the memberships of golf clubs in Taiwan's central region and determine whether golfers' involvement in activities affects the degree of place attachment. They also add two factors of activity experience and experience value to develop a theoretical framework. This theoretical model was based on six hypotheses. The first two were related to how local attachment and activity experience are affected by involvement. The other four hypotheses identified significant relationships among activity experience, experience value, activity involvement, and local attachment. As their outcomes reveal, all hypotheses were supported except for the last one.

The last paper of this special issue is entitled “Examining ownership equity as a psychological factor on tourism business failure forecasting,” authored by Korol and Spyridou. This paper examines ownership equity as a predictor of future business failure within the tourism and hospitality sectors. This study's main goals were to examine which ratios are the most important in a model forecasting failure for tourism businesses. The authors sought to determine if, in a strict financial model, there exist ratios that can be associated from a psychological point of view. Using an expert-driven approach, they demonstrated that experts' judgment is an appropriate way to develop a bankruptcy prediction model. Finally, they conclude that the MAN ratio (total percentage of equity ownership by company directors), which is often considered an important psychological factor, was the fourth most important ratio for developing a bankruptcy model.

## Author Contributions

All authors listed have made a substantial, direct and intellectual contribution to the work, and approved it for publication.

## Conflict of Interest

The authors declare that the research was conducted in the absence of any commercial or financial relationships that could be construed as a potential conflict of interest.
